# Cerebral venous sinus thrombosis in a young patient with ulcerative colitis: A case report

**DOI:** 10.1097/MD.0000000000017428

**Published:** 2019-10-11

**Authors:** Anna Deskur, Iwona Zawada, Wojciech Błogowski, Teresa Starzyńska

**Affiliations:** a Department of Gastroenterology, Pomeranian Medical University, Szczecin; b Department of Internal Medicine, University of Zielona Góra, Zielona Góra, Poland.

**Keywords:** cerebral venous sinus thrombosis, inflammatory bowel disease, ulcerative colitis

## Abstract

**Rationale:**

Cerebral venous sinus thrombosis (CVST) represents one of the most alarming forms of hemostatic abnormalities that may occur in patients with inflammatory bowel diseases (IBDs).

**Patient concerns:**

Here we report a case of a 25-year-old male with ulcerative colitis, who developed such thromboembolic complication during flare of the disease. CVST in our patient was clinically manifested by headache and nausea.

**Diagnosis:**

Angio-magnetic resonance imaging scan of the head revealed segments of contrast filling defects/absence indicating right dural venous sinus thrombosis of the transverse sinus.

**Intervention:**

Immediate treatment with low-molecular-weight heparin has been introduced and led to full remission of symptoms and total recanalization of the thrombotic cerebral regions.

**Outcomes:**

Currently (over 2 years after diagnosis) the patient is in remission of the disease, and no further thromboembolic complications have been observed.

**Lessons:**

Our case study highlights the clinical difficulties and challenges associated with diagnosis and treatment of CVST, as well as presents the current state of knowledge about this complication among patients with IBDs. Physicians taking care of IBD patients should be aware of this alarming hemostatic abnormality.

## 1 Introduction

Inflammatory bowel diseases (IBDs) represent a major clinical problem. According to the recent epidemiological data, IBDs are most prevalent in well-developed western European and North American countries, where reported rates of new cases remain stable, however, are still higher than in less industrialized regions of the world.^[1]^ These diseases most commonly affect young individuals, particularly women in their 2^nd^ or 3^rd^ decade of life, who usually require life-long treatment.^[1–5]^ Unfortunately, in the course of IBD patients experience unpredictable periods of flares, and sometimes develop severe complications that may lead to (permanent) disabilities and life-threatening conditions. These are usually a consequence of multiple alterations in the function of immune cells and abnormal systemic biochemical profile that are observed in patients with IBD. Among the broad spectrum of biochemical changes present in patients with IBD, systemic hemostatic abnormalities are perceived to be an important problem, as sometimes are easily omitted and eventually may dramatically affect the general outcome of affected individuals. Cerebral venous sinus thrombosis (CVST) represents one of the most alarming forms of hemostatic abnormalities that may occur in patients with IBD, and also profoundly contribute to an increased mortality among affect individuals.^[5–8]^ Here we report a case of a 25-year-old male with a 2-year history of ulcerative colitis (UC), who developed such thromboembolic complication. The patient has provided informed consent for publication of his case.

## 2 The case

A 25-year-old male (KJ) with well-controlled asthma, hypertension, and a 2-year history of steroid-dependent UC was admitted to our department because of a flare of IBD. Before admission, the patient was maintained with mesalazine (3 g/day) and steroids (prednisone, 10 mg/day), as our previous attempt to introduce azathioprine into his immunosuppressive protocol has been unsuccessful. Moreover, he did not give consent to the biological therapy with anti-tumor necrosis factor (TNF) alpha antibodies.

Upon admission the patient reported abdominal pain, loose stools with blood and mucus (6 per day), weight loss (15 kg during the last 3 weeks), and pain in his knee joints and lumbar spine. Moreover, he was complaining of severe headache and nausea—a set of symptoms he had been experiencing (with different intensity) since the beginning of the disease, and could not successfully deal with. Because of that he had been consulted by various physicians in the outpatient setting throughout the last 2 years, including a neurologist. He also underwent cervical spine x-ray together with frequent blood pressure measurements to exclude other (than IBD) potential causes of his headache. Repeated physical examinations and results of imaging/biochemical tests revealed no abnormalities. Therefore, his symptoms had been attributed to the general clinical picture of UC, particularly given that this patient was slightly anemic and his headaches usually intensified during flares of UC. The patient had been advised to use analgesics (including tramadol) when needed. However, this approach offered only temporary and modest improvement of his symptoms. Over these two years consulting gastroenterologist(s) attempted to slightly increase the dose of steroids (temporarily up to 45 mg/day), hoping it will put the primary disease under better control, and will result in resolution of patient's symptoms. However, similarly to analgesics this therapy also did not bring any improvement. During the 2 months preceding admission the patient had experienced headache (every day) that was particularly disturbing and woke him up during the night. Upon admission the patient described it as a diffuse, throbbing, and excruciating pain of high intensity (8/10 according to the Visual Analogue Scale—VAS) that was present mainly in the occipital and temporal regions of his head, and was accompanied by nausea, but not vomiting.

Physical examination revealed left-sided abdominal pain. In laboratory tests (presented in detail in Table 1), anemia together with elevation in d-dimers and stool calprotectin levels were observed. Presence of parasites in the stool had been excluded. An immediate consultation by a neurologist had been requested and angio-magnetic resonance imaging (MRI) scan of the head had been performed. Neuroimaging evaluation revealed segments of contrast filling defects/absence suggesting right dural venous sinus thrombosis of the transverse sinus. Immediate treatment with low-molecular-weight heparin (LMWH) in a daily dose of 1.6 mL has been introduced (Enoxaparin 80 mg/0.8 mL s.c.; twice a day). Moreover, colonoscopy was performed and revealed macroscopic features of severe edema, inflammation, and multiple mucosal ulcerations present on a whole length of the colon, together with inflammatory polyps present in the descending and sigmoid colon, as well as in rectum. Flare of UC had been managed with mesalazine, steroids, and azathioprine.

**Table 1:**
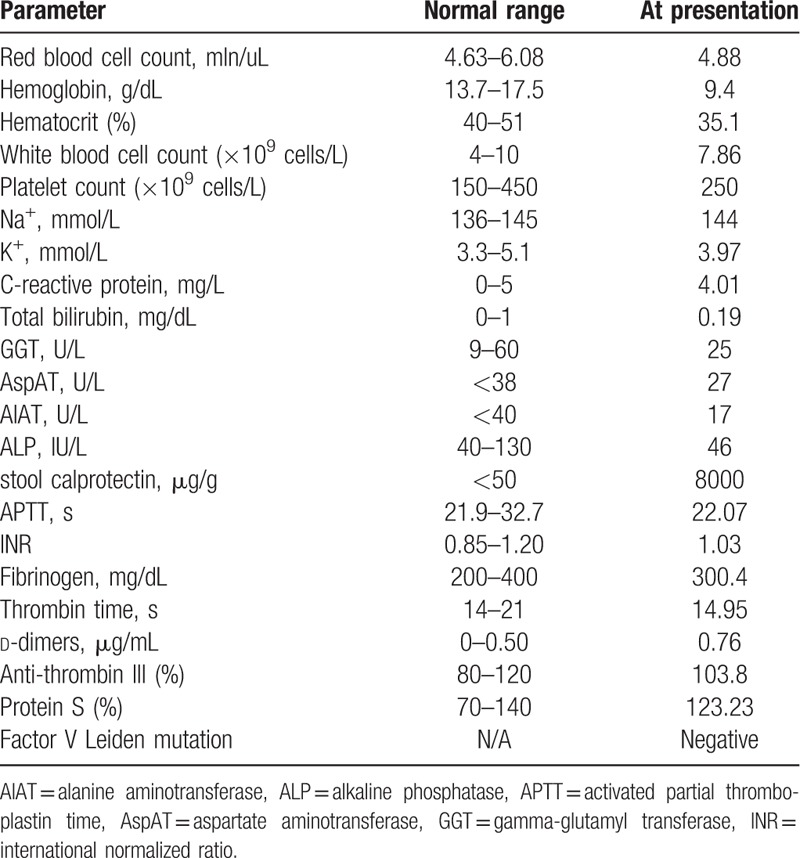
Results of laboratory tests.

Within 2 days following initiation of treatment, the patient stopped feeling headache, and his digestive symptoms had resolved. Further comprehensive evaluation by hematologists revealed no signs of thrombophilia or any other inborn coagulation defects. He was discharged from the hospital on the 25^th^ day with significant improvement. Anticoagulant therapy had been maintained with LMWH (Enoxaparin 80 mg/0.8 mL s.c.; twice a day) for 15 months. Subsequent angio-MRI scans taken after 2 and 5 months following the admission and treatment revealed recanalization of the thrombotic cerebral regions. The patient appears for regular follow-up visits in our outpatient clinic every 4 to 6 months and no longer experiences any headaches.

## 3 Discussion

Hemostatic abnormalities represent a major threat among IBD patients. In comparison to the general population, patients with IBD have around 3-times higher risk of thrombosis. However, those with flare of the disease experience up to even 16-times higher risk of thromboembolic episodes (TE) than healthy population.^[9]^ Unfortunately, as for now there are no specific nor sensitive (predictive) biomarkers available that would allow to successfully identify those patients who are at risk. To make matter worse, it has been demonstrated that around half of IBD patients developing TE have no identifiable risk factor.^[10]^ Therefore, it is of essential value to promote awareness about TE involving cerebral vasculature in patients with IBD.

The general prevalence of all TE among patients with IBD is estimated to be of around 5.6%.^[2]^ In this group of patients, TE most commonly manifests in a form of deep vein thrombosis, pulmonary embolism, sinusoidal, cerebral, or portal vein thrombosis.^[2,4,5]^ These complications usually occur in younger individuals during flares of the disease, and contribute to the (almost 2-times) increased general mortality of affected individuals.^[5–8]^ So far several risk factors that increase the likelihood of TE in IBD patients have been identified, and these include low physical activity or immobility, smoking, obesity, fluid depletion, (recent) surgeries, steroid therapy and inflammation per se.^[11–14]^ Unfortunately, very little is known about the exact molecular mechanisms responsible for the increased risk of hemostatic abnormalities among IBD patients. One may definitively state that in IBD patients several alterations in systemic levels of hemostatic factors are observed, and these create both prothrombotic and hypercoagulative profiles (reviewed in details in ^[15,16]^). It is generally believed that the pathogenesis of these hemostatic alterations is multifactorial, and closely linked to the abnormal (excessive) function of the immune system. For example some studies highlighted potential role of such cytokines as interleukin-6 and TNF-alpha in activation of coagulation among patients with IBD.^[15,17]^ Nonetheless, the exact nature of these interactions is still not precisely defined, particularly in the clinical setting, and remains intensively examined in both experimental and clinical studies.

CVST as manifestation of hemostatic abnormalities in individuals with IBD is very rarely seen. According to recent analyses, it is estimated that it occurs in around 0.5% to 6.7% of all IBD patients, predominantly in young individuals between 2^nd^ and 4^th^ decade of life.^[4–6]^ In relatively mild cases, symptoms presented by patients are usually very nonspecific, and similarly as in the case of our patient, are limited to a headache (reported by around 88%–95% of patients). Other symptoms may include general and/or focal seizures (47%), uni- or bilateral paresis (43%), and/or dysphasia (37%).^[3]^ Presence of (particularly more specific and/or alarming) neurological symptoms usually alerts physicians and motivates them to perform further laboratory and imaging tests. Unfortunately, these are associated with certain limitations when it comes to CVST diagnosis. Namely, recent guidelines of the *European Stroke Organization* are recommending to measure systemic levels of d-dimers in the general population of patients suspected of CVST before neuroimaging tests, unless symptoms are limited only to a headache and/or last longer than for just one week. However, it has been highlighted that the quality of evidence for and strength of these recommendations is inadequate.^[18,19]^ In the context of patients with CVST, a study based on analysis of >70 patients has shown that although elevated levels of d-dimers may be indicative of CVST, still the authors demonstrated that this test lacks adequate diagnostic value to be considered as either sensitive or specific marker of this condition in humans.^[19]^ Unenhanced CT scans have also relatively small diagnostic value for detection of CVST (sensitivity of around 30%), but may be helpful to exclude other (common) reasons that might be responsible for neurologic symptoms presented by the patients (particularly headache). However, CT supported by a contrast agent may reach sensitivity of up to 99%—a value similar to the one observed for MRI, which is considered a criterion standard in detection of CVST (reviewed in detail in^[20]^).

Clinical management that includes prevention and treatment of IBD patients in the context of CVST is quite complex. Generally treatment of IBD patients with TE is similar to this applied in any other groups of patients with thrombosis.^[21]^ It is of major importance to identify those at risk of hemostatic abnormalities and focus on prevention. According to the published consensus by the Canadian Association of Gastroenterology, anticoagulant prophylaxis is generally advised for IBD patients hospitalized because of flares of the disease, as well as for those managed in the outpatient setting with moderate and severe flares of the disease, particularly if there is any history of TE or presence of any aforementioned risk factors.^[22]^ Little is known to what extent such preventive clinical approach is effective. Certain indirect evidences demonstrate that it is generally associated with less frequent TE among IBD patients following hospitalization, and with reduced mortality related to these hemostatic abnormalities.^[23,24]^ In addition, it remains a matter of a debate how long such prophylaxis should be maintained. It is suggested^[22,25]^ that in case of hospitalized patients and/or those with history of TE it should be definitively continued (at least for several weeks) after hospital discharge even if a remission of IBD is achieved (discussed in detail in^[16]^). According to guidelines of the American Heart Association and American Stroke Association, anticoagulative therapy with vitamin K antagonist for 3 to 6 months is recommended in patients with CVST secondary to a transient risk factor, and for 6 to 12 months in those with CVST unrelated to any identifiable risk factor.^[7]^ As for now there are no separate guidelines available regarding treatment of CVST among IBD patients, most likely because of relatively small number of reported clinical cases so far.

In summary, our case study highlights the clinical difficulties and challenges associated with suspicion, diagnosis, and treatment of CVST in IBD patients. Although sometimes CVST may manifest in a form of neurological symptoms, still this and other thrombotic complications should be suspected among IBD patients reporting (particularly long-lasting) headache. Although various laboratory and diagnostic methods have certain limitations, still analysis of d-dimers together with either head CT with contrast agent or MRI remain the optimal method to detect this dangerous complication. Prompt treatment with anticoagulative medications should be continued even after resolution of symptoms and achievement of remission. Careful clinical approach, prompt diagnosis, and adequate treatment remain cardinal determinants of a promising outcome of individuals developing these hemostatic complications. Given the relative difficulty in a timely diagnosis of this rare yet alarming complication, its insidious clinical course, and potentially dramatic consequences, much attention must be paid to promotion of awareness about CVST in patients with IBD, elimination of risk factors associated with it, as well as, to development of various (bio)markers that would help to identify those at particular risk.

## Author contributions

**Conceptualization:** Anna Deskur, Iwona Zawada, Wojciech Blogowski, Teresa Starzynska.

**Investigation:** Anna Deskur, Iwona Zawada, Wojciech Blogowski, Teresa Starzynska.

**Writing – original draft:** Wojciech Blogowski.

**Writing – review & editing:** Anna Deskur, Iwona Zawada, Wojciech Blogowski, Teresa Starzynska.
